# Combined carbapenem resulted in a 4.48-fold increase in valproic acid clearance: a population pharmacokinetic model in Chinese children and adults with epilepsy or after neurosurgery

**DOI:** 10.3389/fphar.2024.1423411

**Published:** 2024-11-08

**Authors:** Luofei Zhang, Ruoyun Wu, Xingmeng Li, Weixing Feng, Zhigang Zhao, Shenghui Mei

**Affiliations:** ^1^ Department of Pharmacy, Beijing Tiantan Hospital, Capital Medical University, Beijing, China; ^2^ Department of Clinical Pharmacology, College of Pharmaceutical Sciences, Capital Medical University, Beijing, China; ^3^ Department of Neurology, Beijing Children’s Hospital, Capital Medical University, Beijing, China

**Keywords:** valproic acid, population pharmacokinetic models, nonlinear mixed effects modeling, epilepsy, carbapenem

## Abstract

Our study aims to explore the pharmacokinetics of valproic acid (VPA) in Chinese patients with epilepsy or after neurosurgery and establish a robust population pharmacokinetics (PPK) model. The PPK model was developed using nonlinear mixed-effects modeling, incorporating a total of 615 VPA plasma concentration data points from 443 Chinese epilepsy or after neurosurgery patients. A one-compartment model with an additive residual model was established. Forward addition and backward elimination strategies were used to assess the impact of covariates on the model parameters. Goodness-of-fit plots, bootstrap, visual predict check and normalized prediction distribution errors were used for model validation. In the final model, the apparent clearance (CL) was estimated using the following formula: $\text{CL }\left(L/h\right)=0.430\times {\left(\text{BW}/60\right)}^{0.787}\times {\left(\text{Cr}/50.3\right)}^{-0.253}\times {\left(\text{ALB}/39\right)}^{-0.873}\times e^{\text{gender}}\times e^{\text{CBP}}{\times e^{\text{IND}2\;}\times e}^{\eta_{\text{CL}}}$ (gender = 0.121 when is female, otherwise = 0; CBP = 1.50 when combined with carbapenems, otherwise = 0; IND2 = 0.15 when combined with oxcarbazepine, carbamazepine, phenobarbital, or phenytoin, otherwise = 0). The volume of distribution (V_d_) was estimated using the formula: $\text{Vd }\left(L\right)=8.66\times {\left(\text{BW}/60\right)}^{0.751}$ . Comedication with carbapenems could increase VPA clearance by 4.48 times, and comedication with oxcarbazepine could enhance VPA clearance by 116%. Besides, creatinine and albumin could affect VPA clearance. Goodness-of-fit plots, bootstrap, visual predict check and normalized prediction distribution showed acceptable data fit, stability, and predictability of the model. In our study, a PPK model was utilized to attain a more comprehensive insight into these variables, improving the accuracy and individualization of VPA therapy in Chinese patients with epilepsy or after neurosurgery.

## 1 Introduction

Epilepsy is a neurological disease characterized by a persistent predisposition to generate spontaneous epileptic seizures (Romoli et al., 2019; Global, 2019), which can result from almost any insult that perturbs brain function, including the effects of neurosurgery (Devinsky et al., 2018). Globally, epilepsy affects approximately 50 million people in diverse age groups, imposing a substantial economic burden on healthcare systems, individuals, and families (Allers et al., 2015).

Valproic acid (VPA) is a first-line, broad-spectrum, antiepileptic drug widely used for treating epilepsy and preventing epileptic seizures after neurosurgery (Romoli et al., 2019; Guo et al., 2022). VPA demonstrates substantial interindividual variability and possesses a narrow therapeutic window, typically ranging from 50 to 100 mg/L (Teixeira-da-Silva et al., 2022) Underdosing VPA can exacerbate epilepsy and fail to adequately prevent epileptic seizures after neurosurgery effectively, while overdosing is linked to a higher risk of adverse effects including nausea, vomiting, sedation, weight gain, leukopenia, and thrombocytopenia (Methaneethorn, 2017; Methaneethorn, 2018; Zang et al., 2022). Consequently, it is important to tailor dosage regimens and ensure the steady-state serum concentrations of VPA (Johannessen Landmark et al., 2020).

A population pharmacokinetics (PPK) modeling approach facilitates the identification of the sources and correlates of pharmacokinetic variability to optimize individual dosing and therapeutic drug monitoring in target patient populations (Kiang et al., 2012; Darwich et al., 2021). To date, numerous PPK studies have explored the impact of demographic factors on the pharmacokinetic variability of VPA in both pediatric and adult populations. Age, body weight (BW), gender, coadministration of antiepileptic drugs (AEDs), VPA dose, CYP2C9 and CYP2C19 genotypes were found to significantly affect VPA pharmacokinetics (Teixeira-da-Silva et al., 2022; Methaneethorn, 2017; Zang et al., 2022; Birnbaum et al., 2007; Vučićević et al., 2009; Ibarra et al., 2013; Ogusu et al., 2014; Lin et al., 2015; Alqahtani et al., 2019; Correa et al., 2008; Ding et al., 2015; Rodrigues et al., 2018; Xu et al., 2018; Shen et al., 2022). Carbapenem, a classic antibiotic agent with a wide spectrum of antimicrobial coverage, are used widely in neurosurgical patients with active seizure disorder, or those who are at a high risk of developing seizures, for its safety especially in central nervous system tolerability (Li et al., 2021). It has been demonstrated that the serum concentration of VPA decreases significantly by 69.1% with ertapenem and by 65.2% with meropenem, increasing the risk of uncontrolled seizures in patients (Chen et al., 2021). Nonetheless, to date, no PPK model has acknowledged carbapenems as a significant covariate affecting VPA clearance. Besides, Previous research focused exclusively on carbamazepine (CBZ), phenobarbital (PB), and phenytoin (PHT), which are enzyme-inducing AEDs, and analyzed their comedication with VPA. Given that oxcarbazepine (OXC) is a known modest inducer of CYP3A4, our study incorporated OXC among the enzyme-inducing AEDs for analysis (Zaccara and Perucca, 2015; Preuss et al., 2023).

This study aims to explore the pharmacokinetic characteristics of VPA in Chinese patients with epilepsy or after neurosurgery and to develop a robust VPA PPK model for optimizing current VPA therapeutic drug monitoring.

## 2 Methods

### 2.1 Patients and data collection

This study was approved by the Ethics Committee of Beijing Tiantan Hospital, Capital Medical University (KY 2022-018-02) and Beijing Children’s Hospital, Capital Medical University (2019-K-150). Informed consent was obtained from the patients and parents for all participating children or legal guardians in accordance with the guidelines of the Declaration of Helsinki.

The PPK model for VPA was developed based on data from 443 patients (65.5% adults and 34.5% children) with epilepsy or after neurosurgery who were hospitalized in Beijing Tiantan Hospital/Beijing Children’s Hospital affiliated to Capital Medical University between October 2016 and June 2022. Inclusion criteria: 1) patients treated with VPA for epilepsy or after neurosurgery; 2) patients who received regular administration of VPA for at least 1 month; 3) therapeutic drug monitoring was performed during treatment and at least one VPA concentration was obtained. Exclusion criteria: 1) incomplete data records; 2) poor adherence to medication.

The patients were orally administered VPA once or twice daily in the form of solution or sustained tablet form at varying doses ranging from 2.79 mg/kg to 109.09 mg/kg per day. A comprehensive set of data was recorded from the included patients: 1) demographic data: sex, age, BW, height; 2) detailed dosing regimen for VPA: date, time, daily dose, frequency, sampling time; 3) biological data: alanine aminotransferase, aspartate aminotransferase, creatinine (Cr), albumin (ALB); 4) pharmacological data: VPA plasma concentration; 5) concomitant medications: lamotrigine, levetiracetam, CBZ, PB, PHT, OXC, clonazepam, nitrazepam, topiramate, lacosamide, CBP, enzyme inducers (IND1, use at least one of CBZ, PB, and PHT), enzyme inducer (IND2, use at least one of OXC, CBZ, PB, and PHT).

### 2.2 Plasma concentration of VPA

VPA serum concentrations were obtained from therapeutic drug monitoring data of patients, with most of the samples exhibiting steady-state trough concentrations. The total plasma concentrations of VPA were assessed by a fluorescence polarization immunoassay (Centau XP, Siemens, USA), with a quantitative range of 1–150 mg/L. Calibrators and quality control samples were routinely analyzed according to the manufacturer’s quality control instructions.

### 2.3 Statistical analysis

SPSS 27.0 (IBM, Armonk, NY, USA) was used to perform statistical analyses. All the data are reported as the mean ± standard deviation (SD) with median (range).

### 2.4 Development of the population pharmacokinetic model

Phoenix NLME software (version 8.3; Certara, St. Louis, MO) was used to apply a nonlinear mixed-effect modeling methodology. First-order conditional estimation with the extended least squares method (FOCE-ELS) was used to estimate the parameters and variability. Model comparison was performed according to the differences in the objective function value (OFV), Akaike information criterion (AIC) and Bayesian information criterion (BIC). To evaluate the stability and predictive performance of the final model, visual prediction check (VPC), bootstrap analysis, and normalized prediction distribution errors (NPDE) were performed.

#### 2.4.1 Base model

For the base structural model, a one-compartment model with first-order absorption and elimination was performed to describe the pharmacokinetic characteristics of VPA. The model was parameterized using the absorption rate constant (K_a_), V_d_, and CL. Due to the lack of available data during the absorption phase, K_a_ was fixed at 0.46 and 2.64 h^-1^ for the sustained tablets and solutions, respectively, according to previous studies (Ding et al., 2015). The model is described with the following equations (Equations 1–3):

$$dA_{a}/\text{dt}=-K_{a}\times A_{a}$$ (1)

$$dA_{c}/\text{dt}=K_{a}\times A_{a}-CL_{c}\times C_{c}$$ (2)

$$C_{c}=A_{c}/V_{d}$$ (3)

K_a_ and CL_c_ represent the absorption rate and clearance of VPA, respectively. A_a_ and A_c_ represent the amount of VPA in the absorption site and central compartment, respectively. C_c_ represents the VPA concentration in the central compartment.

The interindividual variability (η) of the pharmacokinetic parameters was testedusing an exponential model. Different types of residual variability (ε), including additive, proportional, and exponential models were tested with the aim of finding an optimal model.

#### 2.4.2 Covariate model

Based on the basic model, the impact of various covariates on VPA pharmacokinetic variability was further explored by using stepwise forward inclusion (*p* < 0.01) and stepwise backward deletion (*p* < 0.001). A reduction in OFV of 6.64 (*p* < 0.01) for forward addition and an increase of 10.83 (*p* < 0.001) in OFV for backward elimination were the criteria for retaining covariates in the final model.

#### 2.4.3 Goodness-of-fit and model evaluation

To evaluate the stability, robustness, and predictive ability of the final model, goodness-of-fit plots, bootstrap, VPC and NPDE were generated.

Several scatter plots were utilized to assess the goodness of fit between the base and final models, as follows: 1) observed concentration (DV) versus population predicted concentration (PRED); 2) conditional weighted residuals (CWRES) versus PRED; 3) CWRES versus time after dose (TAD); 4) CWRES versus standard normal quantiles. To evaluate the robustness of the model, a bootstrap (1,000 runs) was performed by resampling the same dataset. The median of the parameters was compared with the estimates of the final model. VPC analysis calculated 5%–95% prediction intervals of the simulated data and compared them with the distributions of the observed values. NPDE (Monte Carlo simulation n = 1,000) was calculated to assess the predictability of the model. Three tests were used to evaluate the final model: 1) the Wilcoxon signed rank test for the mean; 2) Fisher’s test for variance; 3) the Shapiro-Wilks test for a normal distribution. Moreover, a histogram of NPDE, a quantile-quantile plot of NPDE, NPDE versus TAD, and NPDE versus PRED were generated for graphical diagnostics related to NPDE. The data analysis and visualization tasks were carried out using R software (version 4.2.3, https://www.r-project.org).

### 2.5 Simulations for dose selection

Monte Carlo simulations (n = 1,000) were utilized to predict plasma VPA concentrations in patients with typical characteristics under various dosing schedules. Each simulation followed a 24-h dosing interval. The therapeutic range for VPA is defined as 50–100 mg/L (Fleming and Chetty, 2006). Accordingly, recommended dosing regimens tailored to specific types of patient profiles have been outlined to attain this target range.

## 3 Results

### 3.1 Characteristics of enrolled participants

A total of 615 VPA plasma concentrations were obtained from 443 patients (290 females and 153 males) with epilepsy or after neurosurgery in Beijing Tiantan Hospital and Children’s Hospital affiliated to Capital Medical University. Only 50.11% of the patients received VPA monotherapy, and levetiracetam, OXC, and lamotrigine were the most commonly used AEDs in combination. The demographic characteristics, laboratory test results, and coadministered AEDs of the patients are presented in Table 1.

**TABLE 1:** Summary of patient demographic and clinical characteristics.

Characteristic	NO. Of. Subjects = 443 NO. Of. Observation = 615
Demographic data	
Sex (Male/Female)	153/290
Age (years)	32.74 (0.27–84.38) (31.93 ± 22.57)
Body weight (kg)	60.00 (5.50–120.00) (55.15 ± 25.73)
Height (cm)	162.00 (54.00–185.00) (149.80 ± 31.16)
Dosing regimen data	
VPA daily dose (mg/day)	800.00 (80.00–3,000.00) (763.09 ± 396.80)
TDD BW ratio (mg/kg/day)	14.81 (2.79–109.09) (15.71 ± 8.42)
Biological data	
ALT (U/L)	16.20 (0.00–436.20) (23.35 ± 30.15)
AST (U/L)	29.10 (3.10–752.10) (25.49 ± 38.33)
Cr (μmoI/L)	50.30 (13.00–447.60) (52.03 ± 33.67)
ALB (g/L)	39.00 (23.5–51.80) (38.61 ± 4.87)
Pharmacological data	
VPA Concentration (mg/L)	63.01 (0.00–165.05) (62.73 ± 30.81)
NO. of. BLQ concentrations	10 (1.6%)
Concomitant medications	
LTG	28 (6.3%)
LEV	137 (30.9%)
CBZ	15 (3.3%)
PB	23 (5.1%)
PHT	2 (0.4%)
OXC	63 (14.2%)
CZP	26 (5.8%)
NZP	2 (0.4%)
TPM	25 (5.6%)
LAC	13 (2.9%)
CBP meropenem ertapenem	22 (4.9%) 18 (4.1%) 3 (0.6%)
IND 1	35 (7.9%)
IND 2	91 (20.5%)

Data are expressed as median (range) (mean ± SD) or n (%).

TDD, total daily dose; BW, body weight; ALT, alanine aminotransferase; AST, aspartate aminotransferase, Cr creatinine; ALB, albumin; VPA, valproic acid; BLQ, below the limit of quantifcation; LTG, lamotrigine; LEV, levetiracetam; CBZ, carbamazepine; PB, phenobarbital; PHT, phenytoin; OXC, oxcarbazepine; CZP, clonazepam; NZP, nitrazepam; TPM, topiramate; LAC lacosamide; CBP, carbapenems, Enzyme inducer one (including one or more than one of PB, PHT, CBZ), Enzyme inducer two (including one or more than one of PB, PHT, CBZ, OXC).

### 3.2 Development of population pharmacokinetic model

A one-compartment model with first-order absorption and elimination best described the VPA data. The impact of BW on V_d_ was considered when constructing the base model of VPA (Equation 4). However, sparse data hindered the estimation of interindividual variability in V_d_, resulting in large shrinkage that needs to be fixed at zero (Savic and Karlsson, 2009). The OFV of 6,215.05 for the one-compartment model was indicated by the preliminary analysis of the base model. Random residual variability was best described by the additive model.

$$\text{Vd}=\text{TVV}\times \left(\text{BW}∕{\text{BW}}_{\text{Median}}\right)$$ (4)

where TVV is the population typical value of V_d_ (L).

The model development procedures for the final model are shownin Table 2. The results indicated that BW, CBP, ALB, Cr, gender, and IND2 had significant effects on VPA CL and were included in the final model.

**TABLE 2:** Results in the model development procedure of final model.

Model no.	Model description	OFV	∆OFV	*p*-Value
Forward addition				
1	Base model	6,200.62		
2	Add BW on CL in model 1	5,911.08	289.54	<0.01
3	Add Mem on CL in model 2	5,690.50	220.58	<0.01
4	Add ALB on CL in model 3	5,657.51	32.99	<0.01
5	Add Cr on CL in model 4	5,631.61	25.90	<0.01
6	Add gender on CL in model 5	5,619.21	12.40	<0.01
7	Add enzyme inducer 2 on CL in model 6	5,605.18	14.03	<0.01
Backward elimination				
8	Remove BW on CL from model 7	5,899.82	294.64	<0.001
9	Remove Mem on CL from model 7	5,836.44	231.26	<0.001
10	Remove ALB on CL from model 7	5,656.63	51.45	<0.001
11	Remove Cr on CL from model 7	5,633.11	27.93	<0.001
12	Remove gender on CL from model 7	5,617.32	12.14	<0.001
13	Remove enzyme inducer 2 on CL from model 7	5,619.19	14.01	<0.001

ALB, albumin; BW, body weight; CBP, carbapenems, Cr creatinine; CL, clearance, Enzyme inducer two (including one or more than one of PB, PHT, CBZ, OXC), OFV, objective function value.

The estimated apparent distribution volume (V/F) and apparent clearance (CL/F) of VPA for the base model were 19.14 L and 0.44 L/h, respectively. The interindividual was best described by an exponential association, whereas the residual variability was best explained by an additional association.

The final model included Ka, V/F, and CL/F, as shown below (Equations 5, 6):

$$\text{CL }\left(L/h\right)=0.430\times {\left(\text{BW}/60\right)}^{0.787}\times {\left(\text{Cr}/50.3\right)}^{-0.253}\times {\left(\text{ALB}/39\right)}^{-0.873}\times e^{\text{Gender}}\times e^{\text{CBP}}{\times e^{\text{IND}2}\times e}^{\eta_{\text{CL}}}$$ (5)

$$\text{Vd }\left(L\right)=8.66\times {\left(\text{BW}/60\right)}^{0.751}$$ (6)

In the final model, K_a_ was fixed at 0.46 and 2.64 h^-1^ for the sustained tablets and solutions, respectively (Teixeira-da-Silva et al., 2022; Methaneethorn, 2017; Ding et al., 2015). Where 0.430 (L/h) is the typical value of CL (L/h), and 8.66 (L) is the typical value of V_d_ (L). The median values of BW, Cr, and ALB were 60.0 kg, 50.3 μmoI/L, and 39.0 g/L, respectively. The estimated correlation coefficient was 0.787 for CL and BW, and 0.75 for V_d_ and BW. The value of gender was assigned as 0.12 for female and 0 for male. Additionally, the value of CBP was assigned as 1.50 when CBP was coadministered, and it was set to 0 in other cases. And when coadministered with at least one of the enzyme inducers (CBZ, PB, PHT, and OXC), the value of IND2 was assigned as 0.15, otherwise, it would be set to 0. Comprehensive detailed information on the parameter estimates, relative standard errors, 95% confidence intervals, interindividual variability, residual variability, and bootstrap results is displayed in Table 3.

**TABLE 3:** Parameter estimates of the base model, final model and bootstrap analysis.

Parameter	Base model		Final model		Bootstrap	
	Estimate (%RSE)	95% CI	Estimate (%RSE)	95% CI	Median (%RSE)	95% CI
Ka (Solutions) (h^−1^)	2.64	—	2.64	—	2.64	—
Ka (Sustained Tablets) (h^−1^)	0.46	—	0.46	—	0.46	—
V_d_ (L)	19.14 (18.77)	(12.09, 26.20)	8.66 (9.18)	(7.10, 10.22)	8.66 (9.52)	(7.11, 10.39)
CL (L/h)	0.44 (6.40)	(0.39, 0.50)	0.43 (3.22)	(0.40, 0.46)	0.43 (3.33)	(0.41, 0.46)
BW on CL (L/h)	—	—	0.79 (5.99)	(0.69, 0.88)	0.79 (6.06)	(0.69, 0.88)
Gender on CL (L/h)	—	—	0.12 (30.25)	(0.049, 0.19)	0.12 (27.35)	(0.057, 0.19)
Cr on CL (L/h)	—	—	−0.25 (23.50)	(-0.37, −0.14)	−0.26 (24.36)	(-0.38, −0.14)
ALB on CL (L/h)	—	—	−0.87 (14.53)	(-1.12, −0.62)	−0.88 (14.21)	(-1.13, −0.64)
Mem on CL (L/h)	—	—	1.50 (9.79)	(1.21, 1.79)	1.65 (35.25)	(1.24, 4.00)
Enzyme inducer 2 on CL (L/h)	—	—	0.15 (27.81)	(0.067, 0.23)	0.15 (25.51)	(0.073, 0.22)
IIV(exponential)	67.22%	—	23.33%	—	23.08%	—
σ(additive)	25.62 (7.1)	(22.01, 29.22)	17.68 (6.63)	15.38–19.98	17.52 (6.84)	(15.13, 19.69)

Ka absorption rate constant, V_d_ apparent distribution volume, CL, apparent clearance; BW, body weight, Cr creatinine; ALB, albumin, Mero meropenem; IIV, inter-individual variability.

### 3.3 Goodness-of-fit and model evaluation

**FIGURE 1:**
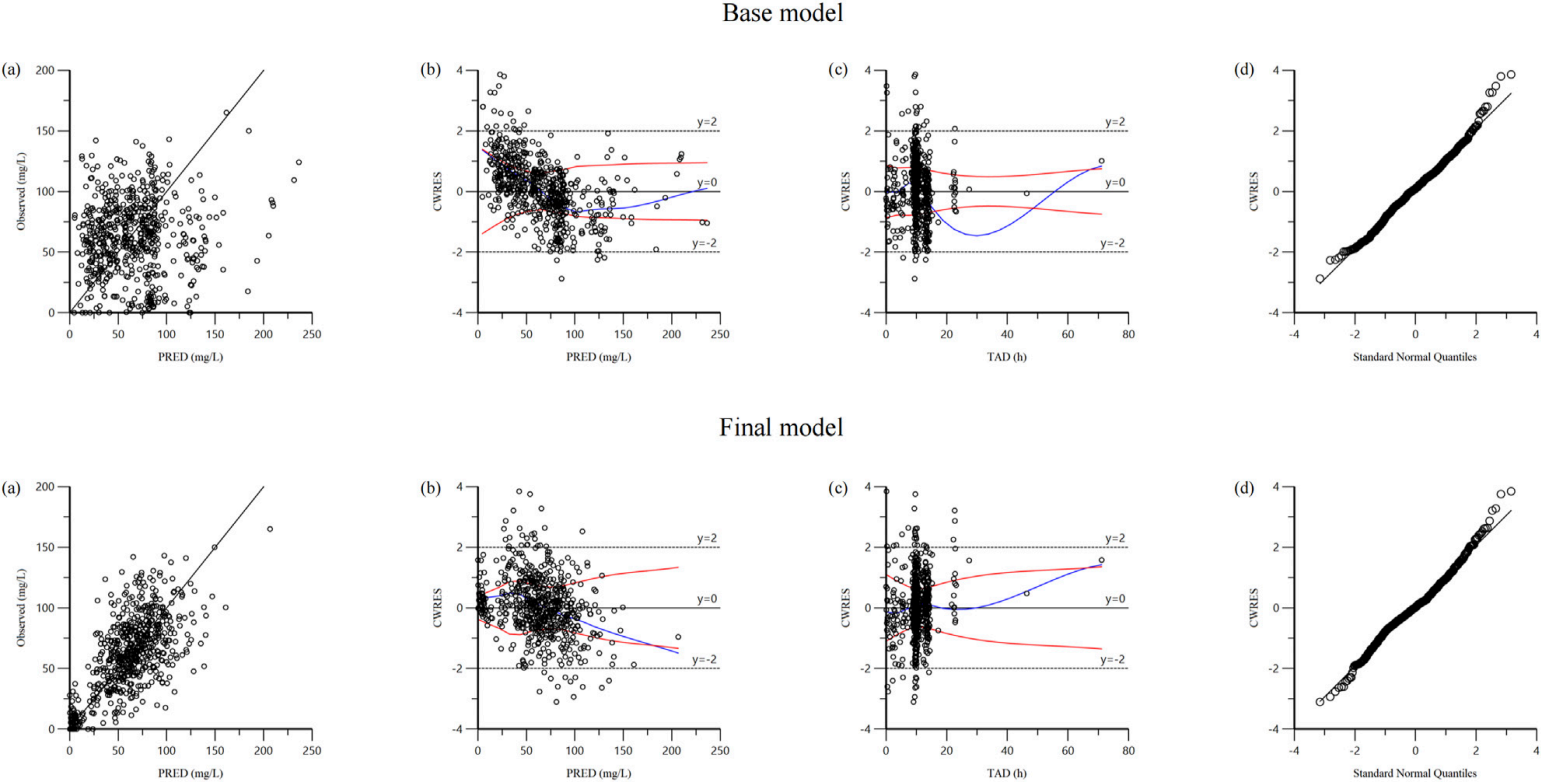
Diagnostic goodness-of fit plots of base model and final model: **(A)** observed versus population predicted concentration (PRED); **(B)** conditional weighted residual (CWRES) versus PRED; **(C)** CWRES versus time after dose (TAD); **(D)** quantile–quantile plots of CWRES.

The reliability of the base model and final model was assessed using goodness-of-fit plots (Figure 1). Compared to the base model, the final model substantially improved the fit of the data. The robust correlation between the predicted and observed concentrations at the population level is illustrated in Figure 1A. The plots of CWRES versus PRED (Figure 1B) and CWRES versus TAD (Figure 1C) demonstrated a uniform distribution around 0 within ±2, and the final model showed no apparent bias or significant trends when compared to the base model. The quantile-quantile plots in Figure 1D indicated that the random effects (η and ε) in the final model adhered to a normal distribution, aligning seamlessly with the underlying modeling assumptions.

The bootstrap analysis with 1,000 runs was conducted for the final model, demonstrating a remarkable consistency and satisfactory results. The parameter estimates of the base model, final model and bootstrap analysis are illustrated in Table 3, indicating that the final models are robust and reproducible.

**FIGURE 2:**
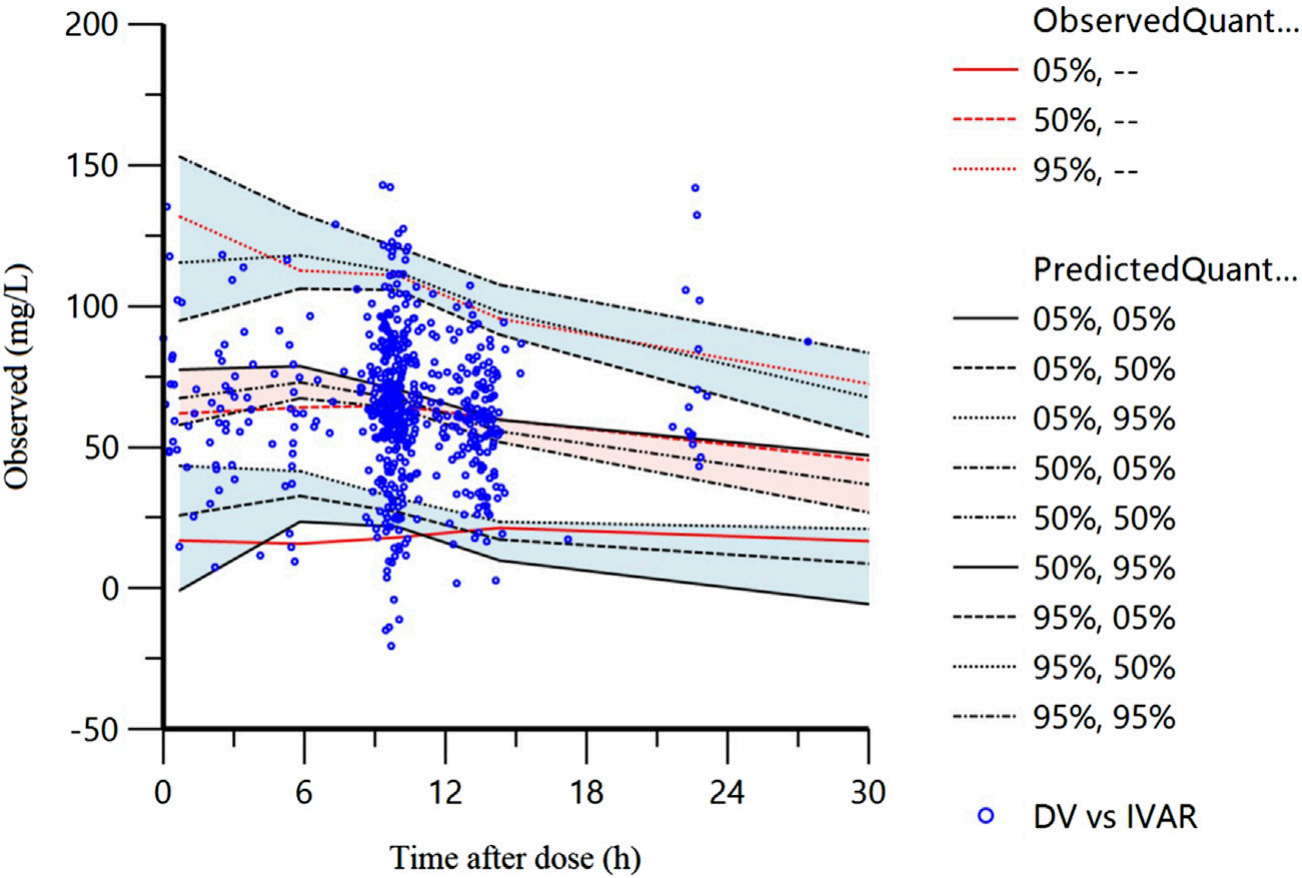
Visual predictive check result of final model.

The VPC of the final model is displayed in Figure 2. The 5%, 50%, and 95% of the observations were distributed approximately within the 95%CI of the simulated concentrations for each interval with 94.1% (579/615) of the measured VPA observed concentrations falling within the 90% prediction interval, suggesting commendable predictive accuracy exhibited by the final model.

**FIGURE 3:**
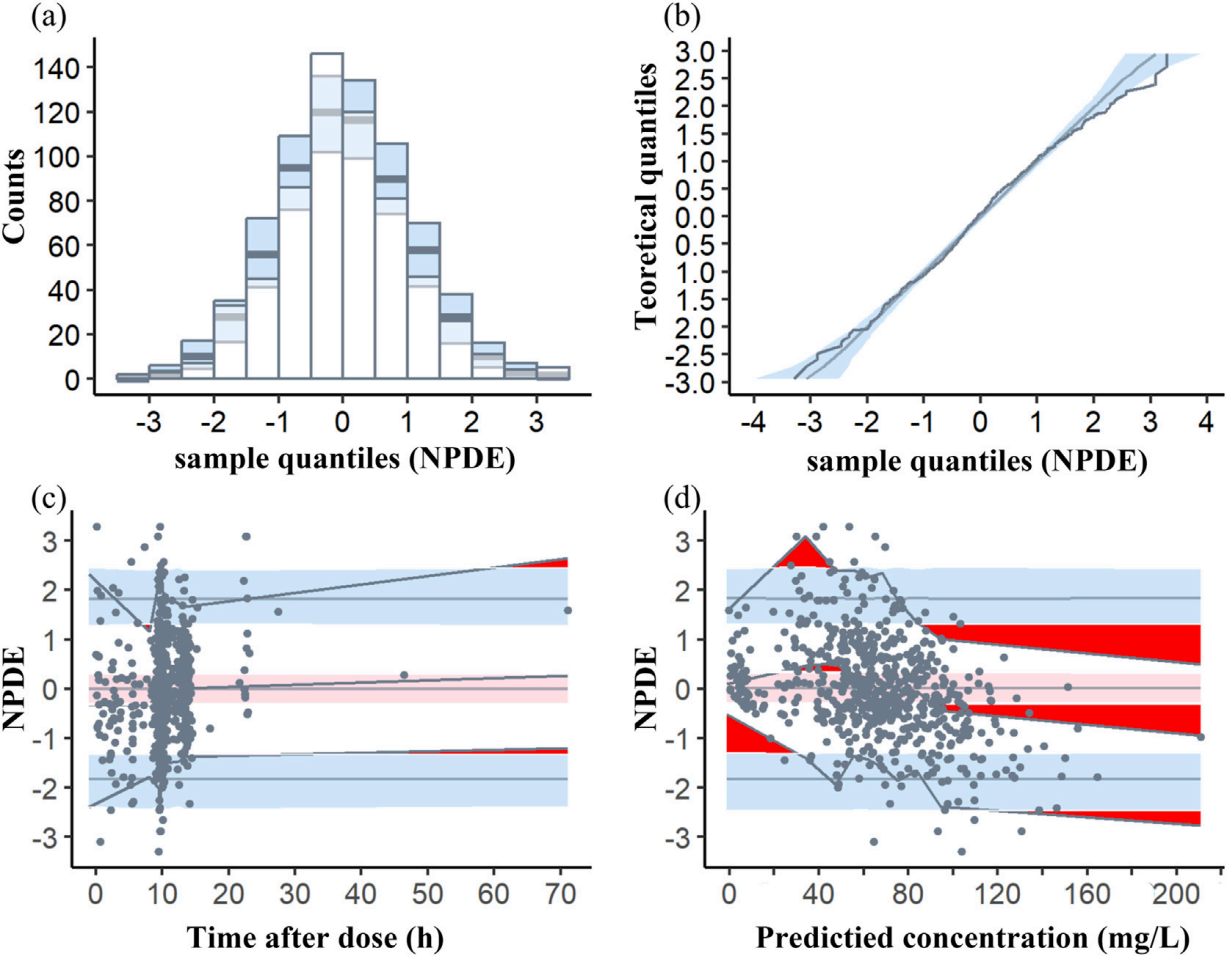
Normalized prediction distribution error (NPDE) plots of the model. **(A)** Histogram of the distribution of the NPDE against theoretical distribution (semitransparent blue fields); **(B)** quantile–quantile plot of the distribution of the NPDE against theoretical distribution (semitransparent blue fields); **(C)** NPDE vs time after dose (h); **(D)** NPDE vs predicted concentrations. In plots **(C, D)**, the three solid black lines represent the actual value of the NPDE at the fifth, 50th and 95th percentiles, the semitransparent red field represents a simulation-based 95% confidence interval for the median at 50th percentile, semitransparent blue fields represent a simulation-based 95% confidence interval for the corresponding model‐predicted 5th and 95th percentiles. The NPDE of the observations are represented by black dots.

The average and variance of NPDE did not exhibit a significant difference from zero (−0.0037, *p*>0.05) and one (1.042, *p* > 0.05), respectively, indicating that the data set did not show a significant difference. Besides, it is noteworthy that the Shapiro-Wilk test results for the final model yielded *p*-values less than 0.01, suggesting that NPDE did not adhere to a normal distribution and may exhibit predictive bias. However, both the quantile-quantile plot and histograms of NPDE values demonstrated a distribution that closely resembled a standard normal distribution, with minimal deviations in relation to time or predicted concentration (Figure 3).

### 3.4 Simulations for dose selection

The simulated doses along with their corresponding 95% confidence intervals (ranging from 2.5% to 97.5%) derived from the final model across various patient categories are represented in Table 4. For instance, if we consider a male weighing 60 kg, aged 33 years, with 50.3 μmoI/L Cr and 39 g/L ALB, not taking CBP and IND2, the suggested dosing regimen for VPA (solutions) would fall within the range of 903 mg (concentration = 50 mg/L) to 1795 mg (concentration = 100 mg/L) every 24 h.

**TABLE 4:** The simulated dose regimen in children with typical characteristics by the final model.

Age (year)	BW (kg)	Dosing interval (h)	ADDL	Dosage form	Cr (μmoI/L)	ALB (g/L)	Gend	CBP	Dose 1 (mg)	Conc 1 (90 %CI) (mg/L)	Dose 2 (mg)	Conc 2 (90 %CI) (mg/L)
Enzyme inducer 2 = NO												
33	60	24	30	1	50.3	39	1	NO	903	50.24 (13.28–93.05)	1795	100.67 (24.08–189.21)
33	75	24	30	1	50.3	39	1	NO	1,095	50.14 (11.16–95.4)	2,149	100.56 (25.18–188.48)
33	60	24	30	1	50.3	39	2	NO	1,089	50.54 (11.46–95.59)	2,203	100.01 (19.04–194.29)
33	75	24	30	1	50.3	39	2	NO	1,301	49.41 (8.83–95.35)	2,660	99.86 (21.69–193.49)
33	60	24	30	1	50.3	39	1	YES	77,200^a^ (avoid concurrent use)	50.52 (-97.74–264.54)	157,200^a^ (avoid concurrent use)	100.37 (-236.6–592.16)
33	75	24	30	1	50.3	39	1	YES	89,950^a^ (avoid concurrent use)	50.52 (-123.28–297.76)	158,200^a^ (avoid concurrent use)	100.36 (-194.98–510.63)
Enzyme inducer 2 = YES												
33	60	24	30	1	50.3	39	1	NO	1,150	50.41 (9.55–97.16)	2,298	100.86 (18.04–196.37)
33	75	24	30	1	50.3	39	1	NO	1,385	49.99 (8.91–98.2)	2,790	100.28 (19.23–192.44)
33	60	24	30	1	50.3	39	2	NO	1,398	49.98 (7.63–98.53)	2,770	99.24 (11.8–200.07)
33	75	24	30	1	50.3	39	2	NO	1700	50.04 (6.51–99.74)	3,600	100.89 (10.02–208.29)

Dosage form 1: solutions; Dosage form 2: sustained tablets; Conc: concentration.

^a^ Concurrent use of VPA, and CBP, is discouraged, and an alternative antibiotic or antiepileptic agent should be considered instead. If coadministration is unavoidable, it is advised to supplement VPA, with another adjunctive AED, during carbapenem treatment and the added medication needs to be continued 7 days after the carbapenem discontinuation.

## 4 Discussion

The PPK model was optimally characterized by a one-compartment model with first-order absorption and elimination, aligning with findings from prior research (Methaneethorn, 2017; Zang et al., 2022; Correa et al., 2008). The typical value of CL was 0.44 L/h, which falls within the previously reported range of 0.206–1.154 L/h (Ogusu et al., 2014; Correa et al., 2008; Ding et al., 2015; Park et al., 2002). Notably, our result closely aligns with the typical CL (0.46 L/h) of Ogusu et al. (Ogusu et al., 2014).This alignment may be attributed to both studies including children and adults, and having similar mean body weights (48.8 kg VS 55.15 kg), as well as shared ethnicity (Asian) across the cohorts. In the final model assessment, relative standard error for all parameters ranged from 3.22% to 30.25%, falling within acceptable limits. Specifically, the coefficient of variation for CL variability was 23.33%, and residual variability was 17.68 mg/L, aligning with prior studies (CV of CL: 13.4%–35.9%, residual variability: 3.11–17.3 mg/L) (Correa et al., 2008; Park et al., 2002; ES et al., 2004). Bootstrap and VPC indicated strong evidence of the model’s stability and predictive capability. However, NPDE analysis fell short of our anticipated standards. Deviating from a normal distribution, the statistical analysis uncovered potential systematic bias in data representation. One tenable hypothesis for this divergence is the paucity of data, which may have engendered inherent biases in the analytical framework. Besides, the Shapiro-Wilk test is highly sensitive to even minor deviations from normality, particularly in large sample sizes, which may lead to the rejection of normality that lacks practical significance. While the majority of the NPDE distributions conform to normality, there may be deviations in the tails that trigger the Shapiro-Wilk test, even when not visually apparent. Subtle skewness or kurtosis may not be easily observed but can still affect the test results.

A lot of studies have found low serum concentrations of VPA during treatment with carbapenems (Šíma et al., 2017; Bates et al., 2010). This model represents the first to investigate the influence of coadministration of carbapenems on CL of VPA. A total of 4.9% of our patients comedicated with carbapenems (4.1% meropenem and 0.6% ertapenem) with VPA. A significant 448.2% increase in VPA CL was observed with comedication involving CBP. Prior studies have investigated the interaction between VPA and carbapenems, indicating that VPA serum concentrations decreased significantly by 69.1% with ertapenem and by 65.2% with meropenem, thereby increasing the risk of uncontrolled seizures in patients (Chen et al., 2021; Huang et al., 2017; Wu et al., 2016; Miranda Herrero et al., 2015). The potential mechanisms of the interaction include inhibition of valproate glucuronide hydrolysis (Torii et al., 2002), induction of valproate hepatic glucuronidation (Torii et al., 2002), increased renal CL of valproate glucuronide and distribution of VPA into red blood cells (Yokogawa et al., 2001). At present, the primary mechanism appears to be the reduced deglucuronidation of valproate glucuronide, attributed to the inhibition of acylpeptide hydrolase by carbapenems (Li et al., 2021; Suzuki et al., 2016). Spriet et al. reported that a patient with severe liver impairment was not affected by the interaction between the two medications (Spriet and Willems, 2011), Zhihong Li et al. found ALT level increased when VPA was concomitantly used with carbapenems (Li et al., 2021). Both studies indicated that the site of interaction was in liver. A total of 36.4% of our patients comedicated with carbapenems had abnormal ALT level higher than the standard values (5–35U/L in women and 5–40 U/L in man), which could possibly be due to the result of the interaction. Concurrent use of these two medications is discouraged and an alternative antibiotic or antiepileptic agent should be considered instead (Hawkey and Livermore, 2012). If coadministration is unavoidable, it is advised to supplement VPA with another adjunctive AED during carbapenem treatment and the added medication needs to be continued 7 days after the carbapenem discontinuation (Li et al., 2021; Huang et al., 2017). On the other hand, certain case reports have suggested that this interaction offers a novel avenue for managing VPA overdose/toxicity (Li et al., 2021; Cunningham et al., 2022).

The interplay between AEDs significantly influences VPA CL. In our final model, concomitant therapy with IND2 notably enhanced VPA CL. Concomitant administration of classic AEDs (CBZ, PB, and PHT) is known to elevate VPA CL in epileptic patients (Teixeira-da-Silva et al., 2022; Ogusu et al., 2014; Lin et al., 2015; Alqahtani et al., 2019). Current research has indicated that potent inducers of cytochrome P450 isozymes or uridine diphosphate glucuronic acid transferase, such as CBZ, PHT, and PB can increase the CL rate of VPA (Ogusu et al., 2014; Correa et al., 2008; Ding et al., 2015). However, only one study has assessed OXC as a covariate but did not identify it as a significant covariate (Zang et al., 2022), probably due to the low rate of drug combination (11.4%) and the weak inducing properties of OXC on CYP3A4 (Zaccara and Perucca, 2015; Preuss et al., 2023).

BW showed a significant impact on V_d_ and CL, aligning with previous PPK models (Zang et al., 2022; Ding et al., 2015; Park et al., 2002). The influence of BW on V_d_ can be principally attributed to variations in fat, lean tissue, and water content among individuals with different BW (Cheymol, 2000). As for CL, the association between BW, an index of body size, and organ development as well as functionality contributes to VPA elimination (Methaneethorn, 2018; Lin et al., 2015).

Additionally, our results firstly demonstrated that VPA CL decreased as Cr or ALB increased, diverging from the previous studies by Zang, Y. N. et al., Jiang et al., and Xu, S. et al., Alqahtani, S. et al. which found no significant impact of Cr or ALB on VPA CL (Zang et al., 2022; Alqahtani et al., 2019; Xu et al., 2018; Jiang et al., 2007). This may be due to the predominantly normal kidney and liver function of the patients in those studies. However, as for kidney function, Cr levels ranged from 13.00 to 447.60 μmol/L, with 33.86% of patients exhibiting impaired renal function (normal renal function: 44.0 to 132 μmoI/L). Approximately 70%–80% of VPA are excreted renally, and patients with renal dysfunction typically present with elevated Cr levels (Bruni and Wilder, 1979). Consequently, patients with renal dysfunction exhibit reduced VPA clearance. As for liver function, a total of 21.90% patients in our study had ALB levels below the standard range (28–54 g/L), which is indicative of abnormal liver function. Given that a total of 90%–95% VPA is bound to plasma proteins (Vučićević et al., 2009; Jankovic et al., 2010), a decrease in ALB levels, leading to more unbound VPA, results in increased VPA clearance and metabolism (Shen et al., 2022).

Our model indicates that women exhibit a 12.9% higher CL of VPA than men. Nonetheless, several studies have observed a 5.7%–37.17% lower VPA CL in women than in men (Zang et al., 2022; Birnbaum et al., 2007; Ibarra et al., 2013; Ogusu et al., 2014), potentially due to greater BW in men or lower Uridine 5′-diphospho-glucuronosyltransferase activity (a major route of VPA metabolism) in women (Methaneethorn, 2017; Ogusu et al., 2014; Court, 2010). Other studies failed to find the influence of gender on VPA CL (Methaneethorn, 2017; Birnbaum et al., 2007; Vučićević et al., 2009; Ibarra et al., 2013; Ogusu et al., 2014; Lin et al., 2015; Correa et al., 2008; Ding et al., 2015; Rodrigues et al., 2018). In our study, the median BW in women was 65 kg (ranging from 7 kg to 110 kg), while in men it was 57 kg (ranging from 5.5 kg to 120 kg), suggesting that a larger body size in women may contribute to an increased capacity for drug elimination (Lin et al., 2015). Although some studies have suggested that the CL difference between sexes could be due to variations in BW, adding BW to the model did not eliminate the inter-individual variability associated with sex (Zang et al., 2022). This indicates that BW differences alone cannot fully explain the CL differences between males and females (Zang et al., 2022). Therefore, both sex and BW were included as covariates.

For concentration simulations, when VPA was administered, the conventional therapeutic daily doses ranged from 500 mg to 2 g for adults or 15–60 mg/kg for children (McNamara, 2001). A total of 45% of the 20 simulated dosing regimens were within the recommended dose range (details in Table 4). When comedicated with carbapenems, in order to achieve the therapeutic window of VPA, oral VPA regimens were from 77,200 to 158,200 mg, approximately 79.1 times higher than typically recommended range, indicating that the concurrent use of carbapenems with VPA is strictly prohibited. Additionally, in order to achieve the prescribed dosage range, patients aged 33 years weighing 75 kg necessitate an additional 200 mg of VPA compared to those with a weight of 60 kg. And when comedicated with other AEDs with enzyme-induction effects, patients aged 33 years with 60 kg or 75 kg necessitate an additional 300 mg VPA. Besides, in seven specific regimens, the dosage surpassed the maximum daily dose to reach 100 mg/L. This suggests that the established upper threshold of 100 mg/L for effective concentration may not be suitable for all adults.

Limitations: 1) the analysis utilized a comparatively modest cohort of 443 patients with 615 observations; 2) the variability assessment for V_d_ and K_a_ were constrained by the limited sampling strategy; 3) the lack of external model validation stemmed from the absence of relevant data from peer research entities; 4) the influences of dietary and genetic factors on valproic acid metabolism were not incorporated.

## 5 Conclusion

A robust VPA PPK model was meticulously developed in this study. The identified covariates affecting VPA CL include BW, CBP, ALB, Cr, gender and coadministration of PB, PHT, CBZ, and OXC. The model’s integrity was affirmed through comprehensive analyses, including bootstrap analysis, visual prediction testing, and NPDE analysis, demonstrating its robustness and predictive accuracy. This model might be helpful for individualized VPA therapy in patients with epilepsy or after neurosurgery. Due to various limitations, larger and prospective studies are warranted to confirm these results.

## Data Availability

The raw data supporting the conclusions of this article will be made available by the authors, without undue reservation.
